# Suppressor Screen and Phenotype Analyses Revealed an Emerging Role of the Monofunctional Peroxisomal Enoyl-CoA Hydratase 2 in Compensated Cell Enlargement

**DOI:** 10.3389/fpls.2016.00132

**Published:** 2016-02-17

**Authors:** Mana Katano, Kazuki Takahashi, Tomonari Hirano, Yusuke Kazama, Tomoko Abe, Hirokazu Tsukaya, Ali Ferjani

**Affiliations:** ^1^Department of Biology, Tokyo Gakugei UniversityTokyo, Japan; ^2^Department of Biochemistry and Applied Biosciences, Miyazaki UniversityMiyazaki, Japan; ^3^RIKEN Nishina CenterSaitama, Japan; ^4^Department of Biological Sciences, Graduate School of Science, University of TokyoTokyo, Japan; ^5^Okazaki Institute for Integrative Bioscience, National Institutes of Natural SciencesOkazaki, Japan

**Keywords:** *Arabidopsis*, cotyledons, compensation, seed storage lipids, H^+^-PPase, gluconeogenesis, Enoyl-CoA Hydratase 2

## Abstract

Efficient use of seed nutrient reserves is crucial for germination and establishment of plant seedlings. Mobilizing seed oil reserves in *Arabidopsis* involves β-oxidation, the glyoxylate cycle, and gluconeogenesis, which provide essential energy and the carbon skeletons needed to sustain seedling growth until photoautotrophy is acquired. We demonstrated that H^+^-PPase activity is required for gluconeogenesis. Lack of H^+^-PPase in *fugu5* mutants increases cytosolic pyrophosphate (PPi) levels, which partially reduces sucrose synthesis *de novo* and inhibits cell division. In contrast, post-mitotic cell expansion in cotyledons was unusually enhanced, a phenotype called compensation. Therefore, it appears that PPi inhibits several cellular functions, including cell cycling, to trigger compensated cell enlargement (CCE). Here, we mutagenized *fugu5-1* seeds with ^12^C^6+^ heavy-ion irradiation and screened mutations that restrain CCE to gain insight into the genetic pathway(s) involved in CCE. We isolated A#3-1, in which cell size was severely reduced, but cell number remained similar to that of original *fugu5-1*. Moreover, cell number decreased in A#3-1 single mutant (A#3-1sm), similar to that of *fugu5-1*, but cell size was almost equal to that of the wild type. Surprisingly, A#3-1 mutation did not affect CCE in other compensation exhibiting mutant backgrounds, such as *an3-4* and *fugu2-1/fas1-6*. Subsequent map-based cloning combined with genome sequencing and HRM curve analysis identified *enoyl-CoA hydratase 2* (*ECH2*) as the causal gene of A#3-1. The above phenotypes were consistently observed in the *ech2-1* allele and supplying sucrose restored the morphological and cellular phenotypes in *fugu5-1*, *ech2-1*, A#3-1sm, *fugu5-1* *ech2-1*, and A#3-1; *fugu5-1*. Taken together, these results suggest that defects in either H^+^-PPase or ECH2 compromise cell proliferation due to defects in mobilizing seed storage lipids. In contrast, ECH2 alone likely promotes CCE during the post-mitotic cell expansion stage of cotyledon development, probably by converting indolebutyric acid to indole acetic acid.

## Introduction

Organogenesis in multicellular organ(ism)s^1^ involves a coordinated interplay of cell proliferation and differentiation. In contrast to animals, developmental regulation in plants does not usually involve cell death or migration; thus, evaluating the contribution of each of these is relatively easy. However, plants can fine tune growth to increase their fitness, rendering the interpretation of growth dynamics complex, particularly in natural habitats (Kuwabara et al., 2003; Nakayama et al., 2014). Such difficulties are easily overcome under optimal laboratory growth conditions where growth is stable and plant sizes and shapes are highly reproducible, making them an excellent model for organogenesis studies (Tsukaya, 1998, 2002, 2005, 2006, 2008; Anastasiou and Lenhard, 2007; Krizek, 2009; Micol, 2009; Ferjani et al., 2010; Czesnick and Lenhard, 2015).

Control of size is a longstanding issue in developmental biology. To address this issue, we have been using *Arabidopsis thaliana* as a model species to investigate size control mechanisms in leaves. Cell proliferation and expansion activities dynamically change during *Arabidopsis* leaf development (Donnelly et al., 1999; Ferjani et al., 2007; Kazama et al., 2010; Horiguchi and Tsukaya, 2011; Ichihashi et al., 2011). More than a decade ago, we noticed that a decrease in the number of cells (or cell proliferative activity) in a developing leaf can trigger an unusual increase in post-mitotic cell expansion activity and thus cell size, which we called compensation (Tsukaya, 1998, 2002). The leaf-phenotype based large-scale screening that we performed in a wild type (WT) background identified several mutations that differentially affect leaf cell number and size at maturity (Horiguchi et al., 2006a,b). Several compensation-exhibiting mutants have been identified and some of their causal genes have been cloned and functionally characterized (Horiguchi et al., 2005; Ferjani et al., 2007, 2011, 2013a; Kawade et al., 2010, 2013; Hisanaga et al., 2013). The cellular dynamics of compensation in aerial lateral organs has been finely dissected and several rules governing triggering of compensation have emerged (Ferjani et al., 2008, 2010, 2013b; Horiguchi and Tsukaya, 2011; Hisanaga et al., 2015). In addition, compensation has been subclassified into three conventional classes based on post-mitotic cell expansion patterns (Ferjani et al., 2013b). In fact, previous kinematic analyses of cell size dynamics during leaf development show that CCE occurs through three different modes (Ferjani et al., 2007), including class I, when post-mitotic cell expansion rate is enhanced, class II, when the post-mitotic cell expansion period is extended, and class III, when increased cell size occurs during the cell proliferative stage (i.e., before the start of post-mitotic cell expansion; Ferjani et al., 2013b; Hisanaga et al., 2015). Nevertheless, our understanding of compensation is limited to the triggering factors, but the link(s) between cell proliferation defects and enhanced post-mitotic cell expansion remain to be elucidated.

The proton-pyrophosphatase (H^+^-PPase) loss-of-function mutant *fugu5* is a compensation-exhibiting mutant with a unique oblong cotyledon shape (Ferjani et al., 2007, 2011). CCE is strong in cotyledons of *fugu5* mutants, but not in leaves formed at later stages (Ferjani et al., 2011). We reported previously that a decrease in cell number in *fugu5* cotyledons is due to lower *de novo* sucrose production from triacylglycerol (TAG) seed reserves (Ferjani et al., 2011). In short, H^+^-PPase loss-of-function in *Arabidopsis* invokes an increase in cytosolic PPi levels, which likely compromise gluconeogenesis from TAG during germination and seedling establishment (Ferjani et al., 2011, 2012, 2014a,b).

Interestingly, although a lack of sucrose production during germination in *fugu5* mutants suppresses cell cycling in cotyledons, palisade tissue cells within the same organs reach larger sizes (∼1.8-fold) compared to those in the WT (Ferjani et al., 2011, 2014b). Supplying carbon in growth media, such as sucrose or glucose, not only restores cell number, but also cell size in mature cotyledons. In addition, specifically removing PPi by expressing the soluble type PPase IPP1 also restores the *fugu5* phenotype, regardless of the presence of sucrose in the growth media (Ferjani et al., 2011, 2012, 2014b). Why is CCE in palisade tissue cells also suppressed? The above findings strongly suggest that: (1) the lower number of cells or the state of cell proliferation itself is somehow monitored or sensed, and that such cues trigger CCE at later developmental stages. (2) Sucrose is not a limiting factor for CCE. As stated above, the triggering conditions for CCE in *fugu5* are understood in some detail, but the mechanisms mediating CCE remain vague. Thus, we wanted to understand the CCE-mediating factors for *fugu5*.

In the present study, we mutagenized *fugu5-1* seeds with ^12^C^6+^ heavy-ion irradiation, and screened for mutations that either partially or totally restrained CCE. Importantly, we isolated the A#3-1; *fugu5-1* mutant line in which the cell number was similar to that of the original *fugu5-1*, but cell size was significantly reduced. In addition, cell size in A#3-1sm was equal to that of the WT and CCE did not occur. We carried out map-based cloning, whole genome sequencing and HRM curve analyses and a phenotypic characterization of distinct mutant alleles in combination with *fugu5* and other compensation-exhibiting mutants to identify the causal gene in this mutant line. Based on our results, we suggest a working model for regulation of CCE in *fugu5*, where the dual functions of ECH2 likely play a key role in CCE.

## Materials and Methods

### Plant Materials and Growth Conditions

The WT used in this study was *Arabidopsis* Columbia-0 (Col-0) and all mutants had the Col-0 background. The SALK T-DNA homozygous knockout line (SALK_005342c) for At1g75730 was ordered from the *Arabidopsis* Biological Resource Center (Columbus, OH, USA). *ech2-1* mutant allele seeds were a kind gift from Prof. Bonnie Bartel (Department of Biochemistry and Cell Biology, Rice University). Seeds were sown on rockwool (Nitto Boseki, Tokyo, Japan), watered daily with 0.5 g L^–1^ Hyponex solution (Hyponex Tokyo, Japan), and grown under a 16/8 h light/dark cycle with white light fluorescent lamps at approximately 50 μmol m^–2^ s^–1^ at 22°C. Sterilized seeds were sown on MS medium (Wako Pure Chemical, Osaka, Japan) or MS medium with 2% (w/v) sucrose where indicated and solidified using 0.2–0.5% (w/v) gellan gum (Murashige and Skoog, 1962) to determine the effect of medium composition on growth. After sowing the seeds, the MS plates were stored at 4°C in the dark for 3 days. After cold treatment, the seedlings were grown either under light (for cellular phenotype analysis) or in the dark (for analysis of etiolated seedlings) for designated periods of time.

### Microscopic Observations and Phenotypic Analysis

Photographs of plant gross phenotypes were taken with a digital camera (Nikon D5000 Nikkor lens AF-S Micro Nikkor 60 mm; Tokyo, Japan). Leaves were fixed in formalin/acetic acid/alcohol and cleared with chloral solution (200 g chloral hydrate, 20 g glycerol and 50 mL deionized water) to measure leaf area and cell number, as described previously (Tsuge et al., 1996). Whole leaves were observed using a stereoscopic microscope (M165FC; Leica Microsystems, Wetzlar, Germany) equipped with a CCD camera (DFC300FX; Leica Microsystems). Leaf palisade tissue cells were observed and photographed under a light microscope (DM-2500; Leica Microsystems) equipped with Nomarski differential interference contrast optics and a CCD camera. Cell size was determined as mean palisade cell area, detected from a paradermal view, as described previously (Ferjani et al., 2011).

### Seed Irradiation Treatment and Mutant Screening

Dry seeds of the *fugu5-1* mutant were irradiated with ^12^C^6+^ ions using the E5 beam line at the RIKEN RI-beam factory. The detailed irradiation method has been described previously (Kazama et al., 2011; Hirano et al., 2012). The irradiation dose and linear energy transfer (LET) were 300 Gy and 30.0 keV μm^–1^, respectively, under which the highest mutation frequency was acquired (Kazama et al., 2011). M_2_ seeds from 11 M_1_ plants were gathered in each pool and 25 pools were used for mutant screening. In total, 110 M_2_ seeds from each pool were sown on rockwool, which allowed for the identification of A#3-1; *fugu5-1*. The A#3-1; *fugu5-1* mutant line was back-crossed with the WT and the A#3-1sm in the resulting F_2_ population was selected based on its small size, phenotype, and segregation rate. Further evaluation of mutant line phenotype stability was performed in the F_3_ generation prior to use for in-depth analyses.

### Identification of the Mutation Responsible for the A#3-1 Mutant Phenotype

A mapping population was generated by crossing the A#3-1sm mutant with *Arabidopsis* Landsberg *erecta*, as described previously (Ferjani et al., 2011). Genomic DNA extracted from F_2_ plants that showed the A#3-1sm mutant phenotype was selected and subjected to map-based cloning. The A#3-1sm locus was mapped to the lower arm of chromosome 1 using various genetic markers (simple sequence length polymorphism, cleaved amplified polymorphisms, and small insertion/deletions) according to the sequence information available at The *Arabidopsis* Information Resource database^2^.

Genomic DNAs from 40 A#3-1sm plants were extracted using MagExtractor (Toyobo, Osaka, Japan) and the High Pure PCR Product Purification Kit (Roche Diagnostics GmbH, Mannheim, Germany) and pooled. The pooled DNAs were sequenced and candidate mutations are listed as described previously (Hirano et al., 2015). SAMtools (v0.1.16; Li et al., 2009), Pindel (v0.2.4.d; Ye et al., 2009), and the BreakDancer (v1.1; Chen et al., 2009) algorithms with default settings were used after removing the PCR duplicates in the raw data with Picard tools (v1.55^2^) to detect candidate mutations. Two candidate mutations in AT1G75730 and AT1G76150 were identified after rough mapping. An additional 24 F_2_ individuals of the A#3-1sm mapping population were genotyped using HRM curve analysis to further discriminate which of the two mutations was responsible for the A#3-1sm phenotype, as described previously (Kazama et al., 2011). The primer sets (5′-TGT CAG AAC GCA GAC TCC CT-3′ and 5′-CTC CAG CAG ATG AGA GAC TT-3′) and (5′-TGA AGA TTC CCA CGC CCG ATA TTG-3′ and 5′-TCG TGA GGA ATC GTC CAG ATA TGG T-3′) were used. All reactions were performed in duplicate or triplicate in 96-well plates.

### Quantitative Analysis of Total TAGs

The quantities of seed lipid reserves contained in dry seeds and in 1-, 2-, 3-, and 4-days-old etiolated seedlings were measured by determining total TAG using the Triglyceride E-Test assay kit (Wako Pure Chemical). Either 20 dry seeds or 20 seedlings were homogenized in a mortar in 100 μL sterile distilled water. The homogenates were mixed with 0.75 mL of reaction buffer provided in the kit as described previously (Arai et al., 2008; Ferjani et al., 2011). Sample TAG concentration was determined according to the manufacturer’s protocol. Length of etiolated seedlings was determined as described previously (Ferjani et al., 2011).

## Results

### Isolation of A#3-1; *fugu5-1* Mutant with Suppressed CCE

M_2_ seeds were sown on rockwool and putative mutations that affect CCE in *fugu5-1* were screened. A mutation that suppresses CCE in *fugu5* should fulfill the following criteria: the mutation in the *fugu5-1* background should specifically suppress CCE without affecting cell proliferation. Secondly, the function of the causal gene should explain the results unambiguously and should have a potential role in cell expansion.

**FIGURE 1:**
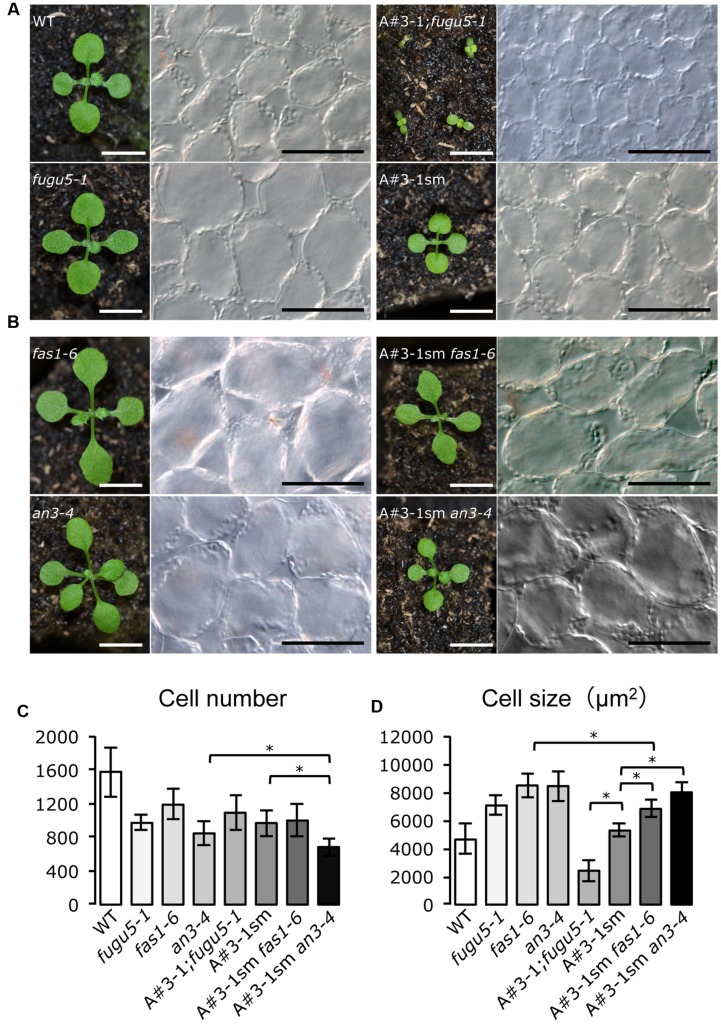
**Isolation of A#3-1; *fugu5-1* mutant with suppressed CCE. (A)** Gross morphology of seedlings (left panels) and images of corresponding palisade tissue cells (right panels) of the WT, *fugu5-1*, A#3-1; *fugu5-*1 and A#3-1sm plants at 13 DAS. **(B)** Gross morphology of *fas1-6*, *an3-4*, A#3-1sm *fas1-6* and A#3-1sm *an3-4* seedlings at 13 DAS. Bar = 5 mm (left panels in **A,B**) or 100 μm (right panels in **A,B**). **(C,D)** Show mean cell number and the size of cotyledon palisade tissue cells of the above lines grown on rockwool for 27 DAS. Data are mean ± standard deviation (*n* = 8). Asterisk indicates significant difference at *P* < 0.05 between the indicated genotypes. DAS, days after sowing.

A candidate mutant should be smaller in size than the WT and *fugu5-1*. Interestingly, among the 17 candidate mutant lines that showed noticeable morphological differences compared to *fugu5-1*, we identified only one mutant line, called A#3-1; *fugu5*, which met the above characteristics. In fact, the A#3-1; *fugu5-1* mutant grew slowly and had oblong cotyledons reminiscent of *fugu5-1*, but overall plant size was significantly reduced (**Figure 1A**). In addition, the gross phenotype of A#3-1sm plants was indistinguishable from WT, except for its small size (**Figure 1A**). Quantifying the number of cells in the cotyledons revealed no differences between *fugu5-1*, A#3-1;*fugu5-1* and A#3-1sm (**Figure 1C**). Importantly, *fugu5-1* exhibited CCE, while cell size in A#3-1sm was indistinguishable from that of WT, and CCE was totally suppressed in A#3-1; *fugu5-1* (**Figures 1A,D**). Taken together, our results indicate that the A#3-1 mutation alone significantly reduced cell number (to the same level as *fugu5-1*), but did not trigger CCE. However, the A#3-1 mutation in the *fugu5-1* background suppressed CCE without affecting cell number, which was already reduced by the *fugu5-1* mutation.

Because the A#3-1 mutation suppressed CCE in *fugu5-1*, which belongs to class II, we were curious whether A#3-1 might also suppress CCE in other classes. Thus, we crossed A#3-1sm to *fugu2-1*/*fasciata*(*fas*)*1-6* and *angutifolia*(*an*)*3-4* mutants of major class I and generated double mutants (**Figure 1B**). Surprisingly, CCE in A#3-1sm *fas1-6* and A#3-1sm *an3-4* were almost unaffected by the A#3-1sm mutation and cell size of mature cotyledons in these lines was similar to *fas1-6* and *an3-4*, respectively (**Figures 1B,D**). These results confirm that CCE in classes I and II are qualitatively different and that the A#3-1 mutation only suppressed CCE in class II compensation.

### Molecular Lesions in the A#3-1; *fugu5-1* Mutant Identified by Map based Cloning, Whole Genome Sequencing and HRM Analyses

**FIGURE 2:**
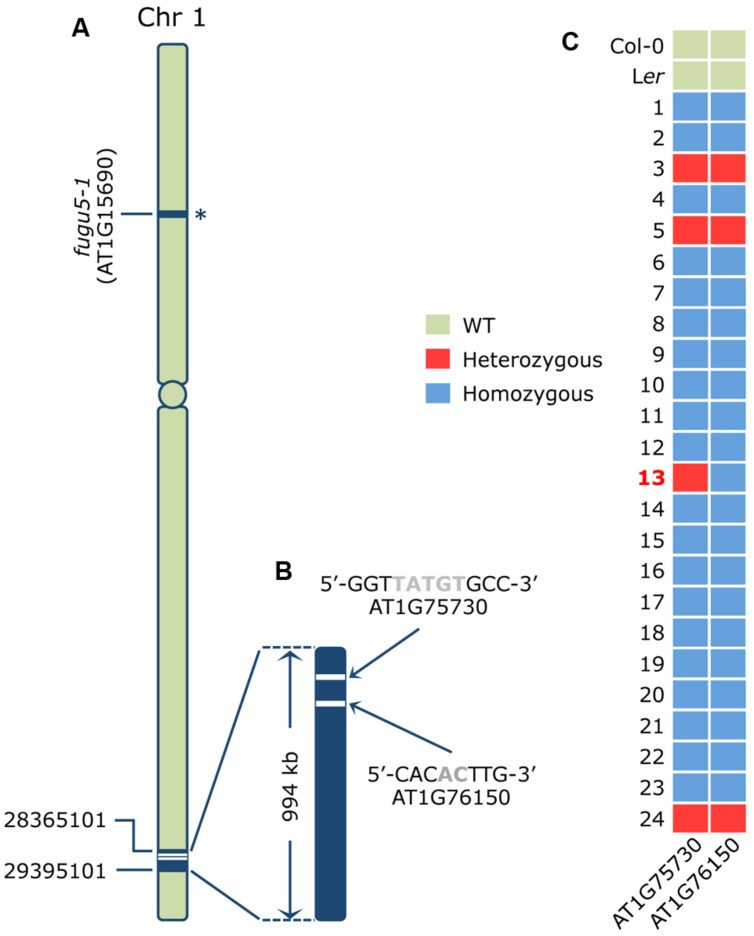
**Identification of the causative mutation in the A#3-1; *fugu5-1* mutant. (A)** Schematic representation of chromosome 1 of *Arabidopsis thaliana* where the *AVP1*/*FUGU5* locus harboring the molecular lesion in the loss-of-function *fugu5-1* allele is indicated by an asterisk. Map-based cloning mapped the A#3-1; *fugu5-1* causative mutation to the lower arm of chromosome 1 between positions 28365101 and 29395101, as indicated. **(B)** Whole genome sequencing data of A#3-1sm genomic DNA and rough mapping identified two mutations within a 994 kb mapping interval. The two candidate mutations were a 5-bp deletion (TATGT at position 28436746-28436750 causing a frame shift) in exon 9 of the AT1G75730 gene encoding a protein of unknown function, and a 2-bp deletion (AC at the 28575546-28575547 position causing a frame shift) in exon 10 of the AT1G76150/*ECH2* gene encoding the monofunctional proxisomal ENOYL-COA HYDRATASE 2. **(C)** To determine which of these two mutations is responsible for the A#3-1 phenotype, an additional 21 F_2_ individual homozygous for the A#3-1sm phenotype and three F_2_ individuals heterozygous for the A#3-1sm phenotype were subjected to genotyping by high-resolution melting (HRM) curve analysis as described previously (Kazama et al., 2011). The nucleotide sequences of the specific primer sets used for HRM are indicated in the Section “Materials and Methods.”

Next, we carried out map-based cloning analyses to identify the causal gene in the *A#3-1;fugu5-1* line. We mapped the causative mutation to a 940 kb fragment in the lower arm of chromosome 1 (**Figure 2A**). Then, we sequenced the whole A#3-1sm genome by NGS and looked for mutations in the mapping interval. The resequence data analyses revealed two homozygous mutations in this region using the Columbia-0 genome as a reference. The first mutation consisted of a 5-bp deletion in exon 9 of the AT1G75730 locus, which encodes a protein of unknown function (**Figure 2B**). The second mutation consisted of a 2-bp deletion in exon 10 of the AT1G76150/*ATECH2*/*ECH2* locus, which encodes for a monofunctional ENOYL-COA HYDRATASE 2. ECH2 is a peroxisomal enzyme that participates in degradation of even *cis*-unsaturated fatty acids (Goepfert et al., 2006) and conversion of indolebutyric acid (IBA) to indole acetic acid (IAA; Strader et al., 2011) and whose gene expression is particularly enhanced during the first 2 days of germination and in senescent leaves.

#### *ENOYL-COA HYDRATASE 2* Gene Mutation Causes the A#3-1sm Phenotypes

Based on the NGS results, A#3-1; *fugu5* was determined to be a triple mutant with mutations in AT1G15690/*AVP1*/*FUGU5*, AT1G75730, and AT1G76150/*ECH2*. The *fugu5-1* mutation was in the upper arm of chromosome 1, whereas the two other mutations were very close (∼140 kb; **Figures 2A,B**). This strong genetic linkage hampered segregation of these two mutations for an individual mutant analysis. Thus, we attempted to validate our results by HRM curve analysis using genomic DNA from the mapping lines confirmed to be either homozygous or heterozygous for the A#3-1sm phenotype (**Figure 2C**). HRM curve analysis is a post-polymerase chain reaction analysis method that accurately discriminates variations in DNA sequences to the single nucleotide resolution level. The method is based on detecting small differences in melting (or dissociation) curves of DNA fragments. As shown in **Figure 2C**, HRM was carried out using genomic DNA from two WT reference genomes (Col-0 and L*er*), 21 mapping lines homozygous for the A#3-1sm phenotype and three mapping lines (#3, #5, and #24) heterozygous for the A#3-1sm phenotype. Importantly, all mapping lines predicted to be either homozygous or heterozygous for the A#3-1sm phenotype were confirmed by HRM to be either homozygous or heterozygous for both mutations in AT1G75730 and AT1G76150, except for mapping line #13. In fact, mapping line #13 plants were homozygous for the A#3-1sm mutation phenotype and for the AT1G76150 mutation, but heterozygous for the AT1G75730 mutation. Taken together, we conclude that a mutation within the AT1G76150/*ECH2* locus provides the A#3-1sm phenotype, rather than AT1G75730.

#### The *ech2-1* Mutant Allele is Indistinguishable from A#3-1sm and Suppresses CCE in *fugu5*

**FIGURE 3:**
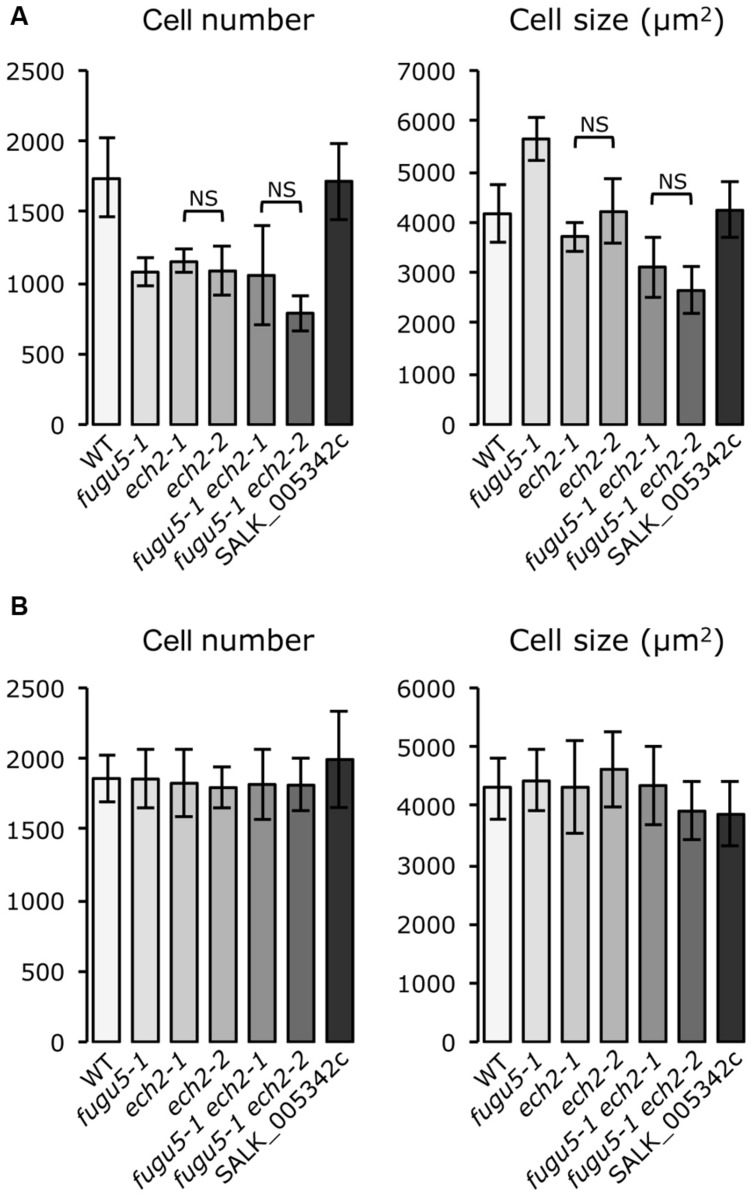
***ech2-2* is the causative mutation that suppresses CCE in a *fugu5-1* background. (A)** Cotyledons from plants grown on rockwool were collected, fixed at 25 DAS and cleared for microscopic analysis. The number and size of palisade cells in the subepidermal layer were determined in the wild type and the indicated mutants. Cell numbers are mean ± standard deviation (*n* = 8). The cell size values are mean cell areas of 160 cells from eight different cotyledons. **(B)** Cotyledons from plants grown on MS media supplemented with 2% sucrose were collected, fixed at 21 days after seed germination and cleared for microscopic analysis. The number and size of palisade cells in the subepidermal layer were determined as described in **(A)**. NS, not significant.

High resolution melting analysis identified *ECH2* as the causal gene of the A#3-1sm mutant. We will refer to A#3-1sm as *ech2-2* and to A#3-1; *fugu5-1* as *fugu5-1 ech2-2*. Although no T-DNA line is available for the *ECH2* locus, an *ech2 RNAi* line (Goepfert et al., 2006) and an *ech2-1* mutant allele harboring a Gly36-to-Glu missense mutation (Strader et al., 2011) have been reported. Strader et al. (2011) reported that an *ech2-1* lesion more severely impairs ECH2 function than the *ech2 RNAi* line. Here, we crossed the *ech2-1* mutant with *fugu5-1*, and all single and double mutants were analyzed for their cotyledon cellular phenotypes to further confirm our findings. Importantly, the gross phenotypes of *ech2-1* and *ech2-2* were indistinguishable (data not shown). The *fugu5-1 ech2-1* and *fugu5-1 ech2-2* double mutants were also consistently indistinguishable (data not shown). In addition, further quantitative analyses of mature cotyledons using these single and double mutants revealed that they had similar cellular phenotypes, confirming the repressive effect of the *ech2* mutation on CCE in the *fugu5-1* background. In contrast, cellular phenotypes of the T-DNA homozygous knockout line (SALK_005342c) for the At1g75730 gene did not differ from the WT (**Figure 3A**). Taken together, these data suggest that CCE in *fugu5-1* is specifically repressed by an additional mutation in the *ECH2* locus.

### Effect of Exogenous Sucrose Supply on CCE

*Arabidopsis* seedlings metabolize fatty acids stored in seeds to fuel growth prior to photosynthesis (Hayashi et al., 1998). *fugu5-1* is dependent on exogenous sucrose to fuel growth following germination and dark grown etiolated seedlings and cotyledon cellular phenotypes are restored after sucrose is supplied (Ferjani et al., 2011). ECH2 is implicated in β-oxidation of even *cis*-unsaturated fatty acids (Goepfert et al., 2006) and the IBA-to-IAA conversion (Strader et al., 2011). Interestingly, several lines of evidence, mainly based on the ability of dark-grown *ech2-1* hypocotyls to elongate normally with or without sucrose, suggest that ECH2 does not mobilize TAG during seed germination and seedling establishment (Strader et al., 2011).

**FIGURE 4:**
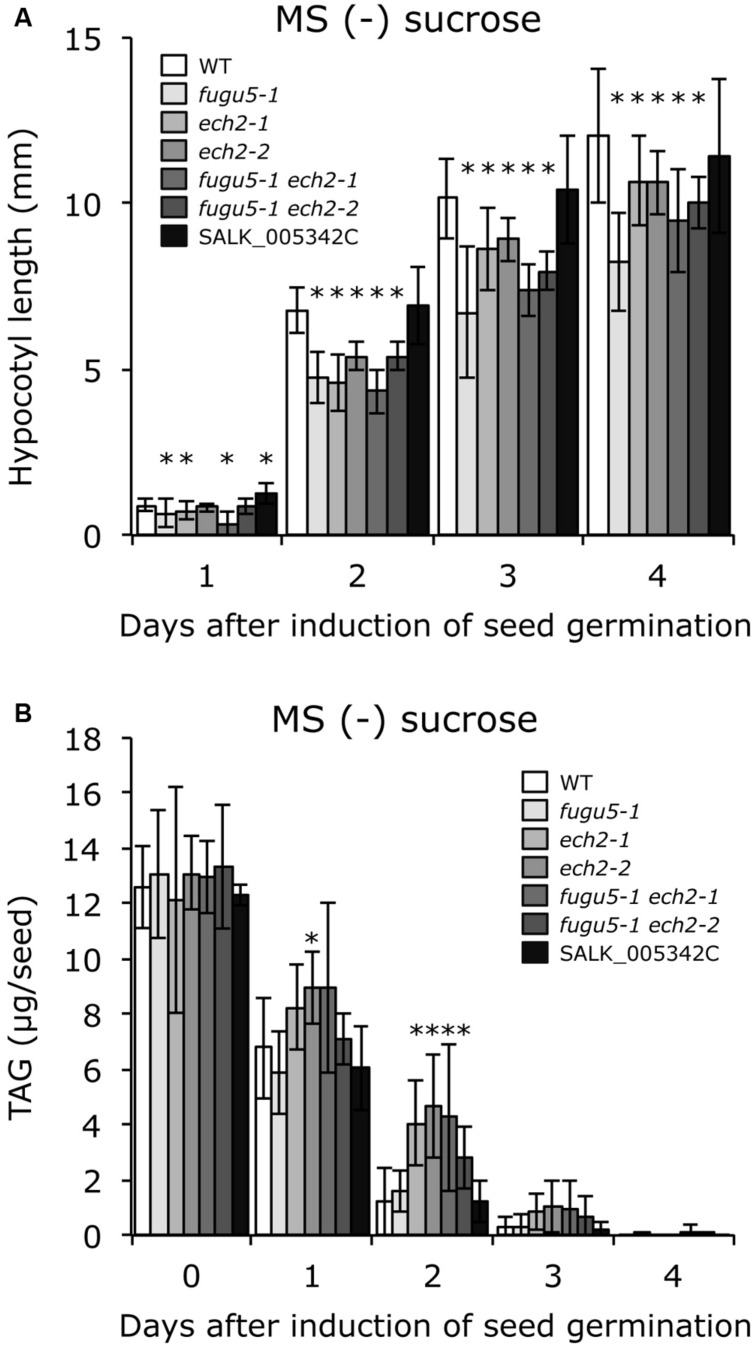
***ech2* mutations impair hypocotyl elongation and mobilization of TAG. (A)** Seeds of the WT and all the mutant lines indicated above were surface sterilized and sown on MS media plates without sucrose. After cold treatment for 3 days at 4°C, the plates were transferred to a growth room (6 h light at 22°C) to induce seed germination. Then, the plates were double covered with aluminum foil and etiolated seedlings were harvested every 24 h (starting from germination) to measure their length. Data were collected from ≥ 17 seedlings at each time. Data are mean ± SD (*n* = 3 independent experiments). Asterisk indicates significant difference at *P* < 0.05 compared to WT. **(B)** Etiolated seedlings were obtained as described in **(A)**. TAG contents were quantified as described in Section “Materials and Methods”; 20 dry seeds or 20 etiolated seedlings were used for each measurement. Data are mean ± SD (*n* ≥ 5 independent experiments). Asterisk indicates significant difference at *P* < 0.05 compared to the WT. TAG, triacylglycerol.

To test whether sucrose affects CCE, cellular phenotypes of plants grown in the presence of 2% sucrose were examined. Interestingly, both cell number and cell size of mature cotyledons in all single and double mutants were restored to WT levels (**Figure 3B**). In addition, the time course effect of sucrose on dark-grown hypocotyls was examined. Consistent with our previous report, *fugu5-1* hypocotyls were significantly shorter compared to WT (**Figure 4A**). Surprisingly, dark-grown *ech2-1* and *ech2-2* hypocotyls tended to be shorter than those of the WT (**Figure 4A**), suggesting that ECH2 and H^+^-PPase play a mutual role mobilizing seed oil.

**FIGURE 5:**
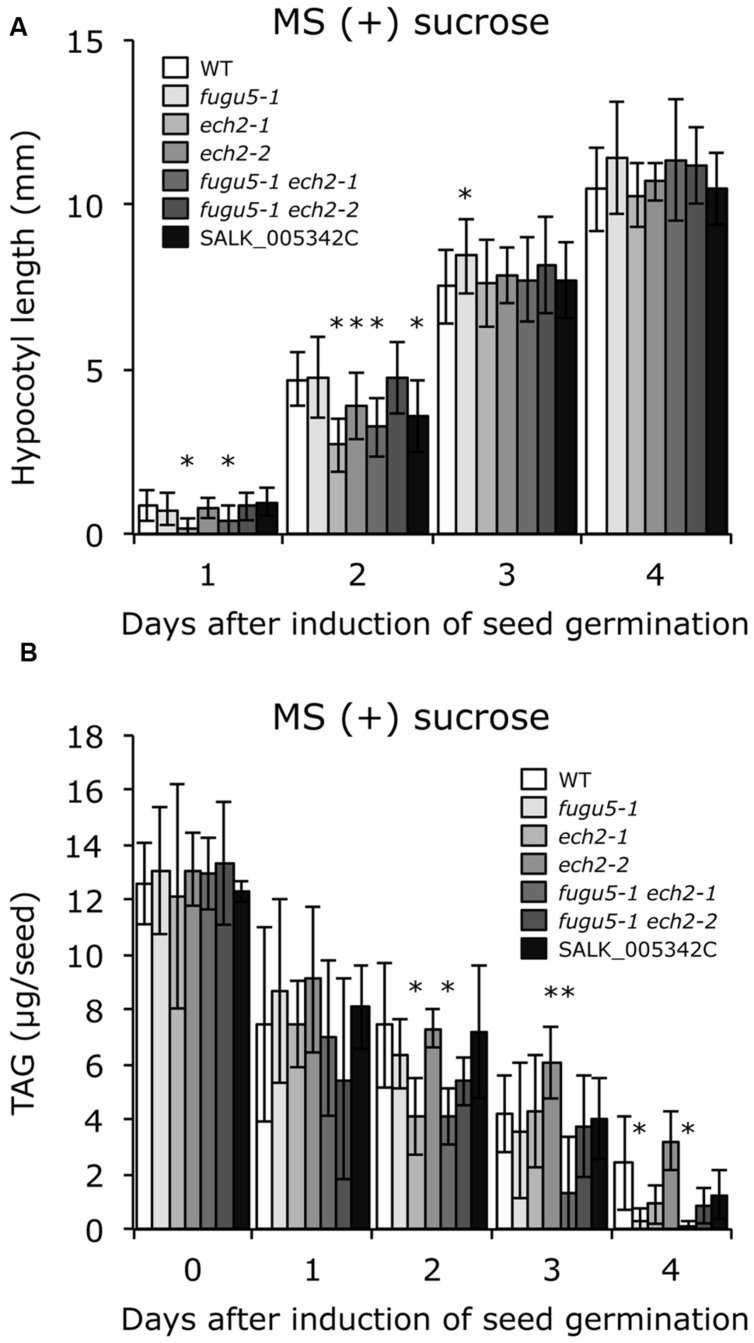
**Exogenous sucrose restores hypocotyl elongation but delays mobilization of TAG regardless of plant genotype. (A)** Seeds of WT and all the mutant lines indicated were surface sterilized and sown on MS medium plates that contained 2% (w/v) sucrose. After cold treatment for 3 days at 4°C, the plates were transferred to a growth room (light for 6 h at 22°C) to induce seed germination. Then, the plates were double covered with aluminum foil and etiolated seedlings were harvested every 24 h (starting from germination) to measure their length. Data were collected from ≥ 14 seedlings at each time. Data are mean ± SD (*n* = 3 independent experiments). Asterisk indicates significant difference at *P* < 0.05 compared to WT. **(B)** Etiolated seedlings were obtained as described in **(A)**. TAG content was quantified as described in Section “Materials and Methods”; 20 dry seeds or 20 etiolated seedlings were used for each measurement. Data are mean ± SD (*n* ≥ 5 independent experiments). Asterisk indicates significant difference at *P* < 0.05 compared to the WT. TAG, triacylglycerol.

Provided that sucrose supply restored the *ech2-1* phenotypes, we next quantified the amount of seed oil reserves in dry seeds and in 1-, 2-, 3-, and 4-days-old etiolated seedlings. TAG mobilization in *fugu5-1* was comparable to that in the WT, and loss of ECH2 function seemed to delay TAG mobilization during etiolated seedling growth (**Figure 4B**). It is noteworthy that hypocotyl elongation was fully restored (**Figure 5A**), whereas TAG mobilization was significantly delayed (**Figure 5B**), regardless of the mutant genotype, when sucrose was supplied exogenously.

## Discussion

### Importance of Peroxisomes for Seed Germination and Seedling Establishment

Peroxisomes fulfill several biochemical reactions of axial importance during plant development, such as the β-oxidation and glyoxylate cycles. In oleaginous plants such as *Arabidopsis*, the β-oxidation cycle is fundamentally important during seedling establishment because it is responsible for breakdown of fatty acids into acetyl-CoA, which is subsequently converted to carbohydrates via the glyoxylate cycle and gluconeogenesis (Gerhardt, 1992; Eastmond et al., 2000; Penfield et al., 2005; Graham, 2008). However, core β-oxidation cannot completely degrade unsaturated fatty acids with *cis* double bonds on even-numbered carbons. Such intermediate products are further processed via the reductase–isomerase and hydratase–epimerase pathways (Kunau et al., 1995). In the latter pathway, unsaturated fatty acids are degraded by core β-oxidation enzymes to 3*R*-hydroxyacyl-CoA, which is converted to 3*S*-hydroxyacyl-CoA before rejoining the core β-oxidation cycle. This conversion is achieved either directly by a 3-hydroxyacyl-CoA epimerase or indirectly by the combined action of ECH2 and ECH1 (for pathway details, see Goepfert et al., 2006, and references therein).

### ECH2 has Dual Functions in Lipid Catabolism and IBA-to-IAA Conversion

ECH2 was originally identified in *Arabidopsis* as a bidirectional enzyme converting 2*E*-enoyl-CoA to 3R-hydroxyacyl-CoA and *vice versa* (Goepfert et al., 2006). *ECH2* mRNA expression is particularly enhanced during the first 2 days of seed germination (Goepfert et al., 2006). Thus, the similarity between expression patterns of *ECH2* and other genes coding for peroxisomal enzymes involved in mobilizing stored lipids, such as *PED1* (Froman et al., 2000), *ICL* (Eastmond et al., 2000) and *MLS* (Cornah et al., 2004), support its involvement in fatty acid catabolism.

Surprisingly, *ech2-1* mutant hypocotyls were previously reported to elongate normally with or without sucrose (Strader et al., 2011), suggesting that ECH2 does not function in β-oxidation during seed germination or seedling establishment (Strader et al., 2011). In contrast, ECH2 is also required for the IBA response (Strader et al., 2011). IBA is an auxin precursor that undergoes peroxisomal β-oxidation to release free IAA, the active form of the plant hormone auxin. Thus, lower IAA content in *ech2-1* seedlings (Strader et al., 2011) and their increased resistance to IBA favored ECH2 in the IBA-to-IAA conversion.

Nevertheless, in the present study, we isolated *ech2-2* as a novel mutant allele and phenotypically characterized it with *ech2-1* to confirm their phenotypes and roles in CCE. Unlike a previous report (Strader et al., 2011), we found that hypocotyl length in plants with the *ech2* alleles decreased significantly, yet to a lesser extent than *fugu5-1* (**Figure 4A**). This reduction in length was associated with delayed catabolism of stored seed lipids (**Figure 4B**). Such shortened hypocotyls cannot be explained by lower IAA levels, as supplying sucrose totally restored *ech2* hypocotyl length. These data indicate that ECH2 has a role in the β-oxidation cycle.

### Cooperative Functions of H^+^-PPase and ECH2 During Seedling Establishment

The *fugu5* mutants fail to sustain heterotrophic growth due to excess cytosolic PPi (Ferjani et al., 2011). High PPi levels partially inhibit gluconeogenesis and reduce hypocotyl length in the absence of an exogenous carbon source (Ferjani et al., 2011). It is believed that cotyledons grow by expanding cells without division after germination (Mansfield and Briarty, 1996). Cotyledon cell number cannot be determined at seed maturity in *Arabidopsis* because after seeds imbibe water the palisade cells undergo an additional round of cell division to double their number reaching ∼1500 cells per cotyledon (Ferjani et al., 2011). This reactivation of cell cycling, which relies on *de novo* sucrose synthesis, was totally suppressed in *fugu5* due to excess PPi, resulting in cotyledons with fewer (∼900 cells), larger cells (Ferjani et al., 2007, 2011). The *ech2* mutants failed to increase cell number in cotyledons because of they failed to efficiently use stored seed lipids. Thus, plants with mutant *ech2* alleles have reduced ability to elongate in the dark for the same reasons. Thus, hypocotyl elongation defects and reduced cell number in cotyledons of both *fugu5-1* and *ech2* can be attributed to the role of their corresponding enzymes in the β-oxidation cycle and gluconeogenesis, respectively.

Although the lack of functional H^+^-PPase in *fugu5* mutants partially reduced *de novo* sucrose synthesis and reduced cell number, these mutants enhanced post-mitotic cell expansion and reached a larger size than WT. Interestingly, while the *ech2* mutations alone decreased cotyledon cell number to the same level as *fugu5*, they failed to enhance post-mitotic cell expansion activity, and *ech2* mutations suppressed CCE in *fugu5*.

### Potential Role of the IBA-to-IAA Conversion in CCE

Although *ECH2* expression peaks during the first 2 days following seed imbibition and in senescent leaves (Goepfert et al., 2006), this does not rule out a role for ECH2 in other organs or during other developmental stages. In fact, *ECH2* is also expressed in green leaves, flowering stems and flowers (Goepfert et al., 2006). Some studies have suggested that IBA-derived IAA promotes root hair elongation and expansion of cotyledon cells. For example, the *abcg36* mutant, which is defective in the putative IBA eﬄux carrier ABCG36 (Strader and Bartel, 2011), has enlarged cotyledons, suggesting that increased accumulation of IBA in these organs increases auxin levels and promotes cell expansion (Strader and Bartel, 2009). Importantly, *abcg36* developmental phenotypes are suppressed when combined with the *ibr1*, *ibr3* and *ibr10* mutations in IBA β-oxidation enzymes INDOLE-3-BUTYRIC ACID RESPONSE1 (IBR1), IBR3, and IBR10, respectively (Zolman et al., 2007, 2008), and the *ibr1 ibr3 ibr10* triple mutant displays small cotyledons (Strader et al., 2010).

In our previous study, we discovered that decreased V-ATPase activity caused by the *de-etiolated*(*det*)*3-1* mutation totally suppresses CCE in a *KRP2* overexpressing (*KRP2* o/e) line, a representative of class III compensation (Ferjani et al., 2013a,b). Consistently, activity of the V-ATPase complex increased significantly in the shoots of *KRP2* o/e plants (Ferjani et al., 2013a). This result indicates that a specific cell cycling defect is apt to unusually increase cell expansion activity (*via* altered protein complex expression and/or activity) and thus promote CCE. Again, in this particular case, the increased V-ATPase activity is likely specific to class III compensation, provided that decreased V-ATPase activity does not suppress CCE in class I (*fas1-6* and *an3-4*) and class II (*fugu5-1*; Ferjani et al., 2013a).

Based on this example, single (*ech2* and *fugu5*) and combined double mutant phenotypes can be unambiguously interpreted if we assume that: (1) failure to mobilize seed stored lipids decreases cell number in cotyledons and somehow enhances ECH2 activity and (2) IAA produced by the action of ECH2 promotes CCE during the post-mitotic stage in *fugu5* cotyledons. Based on these assumptions, the decreased cell number in *fugu5* triggers CCE as described above, yet CCE is suppressed in *fugu5 ech2* double mutants due to the lack of extra IBA-derived IAA. Also, despite the decreased cell number in *ech2* mutants, CCE cannot be triggered because of a lack of additional IBA-derived IAA. Exogenous sucrose is expected to restore cell number and simultaneously suppress the triggering cues to activate ECH2, resulting in normal size cells. In this model, IAA contributes to the extra increase in cell size, but not to the default cell size, as cell size in *ech2* mutants was comparable to that in WT (**Figure 3A**). Although our working model is reasonable, it has to be addressed experimentally in the future for its validation.

### Future Perspectives

More than a decade of hard work on this topic has identified many compensation-exhibiting mutants and cloned and functionally characterized their causal genes, leading to the emergence of basic features of compensation. Compensation occurs in several plant species with loss- or gain-of-function of a particular gene (Horiguchi and Tsukaya, 2011; Ferjani et al., 2012; Hisanaga et al., 2015). In other words, the physiological meaning and significance of compensation in natural habitats has been questioned (i.e., without genetic manipulation). Importantly, we discovered recently that a decrease in ambient temperature induces CCE in palisade tissue cells in North American lake cress, *Rorippa aquatic* (Amano et al., 2015). As the first report of compensation occurring after a fall in ambient temperature, this finding adds value to compensation as a model case to dissect size regulatory networks in plants not only under laboratory conditions, but also in nature.

The major outcome of this study is that the phytohormone auxin may be a candidate molecule to answer some of our longstanding questions about CCE in *fugu5*. Compensation is a heterogeneous phenomenon with different inputs and outputs that differ in each individual mutant that displays CCE (Hisanaga et al., 2015). In fact, as shown here (**Figures 1B,C**), the *ech2-1* mutation did not affect class I compensation mutants, such as *fas1-6* and *an3-4*. Henceforth, CCE suppressor screens for each individual mutant background must be conducted to elucidate the molecular mechanism(s) in such mutants.

## Author Contributions

MK conducted the screening, map-based cloning, HRM analysis and mutant phenotyping and analyzed data; KT performed and confirmed all measurement of cell size and number experiments and analyzed data; YK, TH, and TA performed heavy-ion beam irradiation, NGS data and HRM analyses and contributed to the manuscript. HT, supervised the overall project, contributed to its funding, and to the manuscript drafting. AF, conceived and initiated the project, obtained funding, analyzed the data and wrote the manuscript. All authors read and approved the final manuscript.

## Conflictof Interest Statement

The authors declare that the research was conducted in the absence of any commercial or financial relationships that could be construed as a potential conflict of interest.

## Footnotes

1 http://www.Arabidopsis.org/index.jsp

2 http://picard.sourceforge.net/

## Abbreviations

CCE: compensated cell enlargement
HRM: high resolution melting
NGS: next generation sequencing.
